# Crystal Structure and Activity Studies of the C11 Cysteine Peptidase from *Parabacteroides merdae* in the Human Gut Microbiome*

**DOI:** 10.1074/jbc.M115.706143

**Published:** 2016-03-03

**Authors:** Karen McLuskey, Jaspreet S. Grewal, Debanu Das, Adam Godzik, Scott A. Lesley, Ashley M. Deacon, Graham H. Coombs, Marc-André Elsliger, Ian A. Wilson, Jeremy C. Mottram

**Affiliations:** From the ‡ Wellcome Trust Centre for Molecular Parasitology, Institute of Infection, Immunity and Inflammation, College of Medical, Veterinary and Life Sciences, University of Glasgow, Glasgow G12 8TA, United Kingdom,; the § Department of Biology, Centre for Immunology and Infection, University of York, Wentworth Way, Heslington, York YO10 5DD, United Kingdom,; the ¶ Joint Center for Structural Genomics,; the ‖ Stanford Synchrotron Radiation Lightsource, SLAC National Accelerator Laboratory, Menlo Park, California 94025,; the ‡‡ Department of Integrative Structural and Computational Biology, The Scripps Research Institute, La Jolla, California 92037,; the ‖ Center for Research in Biological Systems, University of California, San Diego, La Jolla, California, 92093,; the ** Program on Bioinformatics and Systems Biology, Sanford Burnham Prebys Medical Discovery Institute, La Jolla, California 92037,; the §§ Protein Sciences Department, Genomics Institute of the Novartis Research Foundation, San Diego, California 92121, and; the ¶¶ Strathclyde Institute of Pharmacy and Biomedical Sciences, University of Strathclyde, Glasgow G4 0RE, United Kingdom

**Keywords:** C-terminal domain (carboxyl tail domain, CTD), crystal structure, cysteine protease, enzyme, proteolysis, active site, domain, kinteoplast

## Abstract

Clan CD cysteine peptidases, a structurally related group of peptidases that include mammalian caspases, exhibit a wide range of important functions, along with a variety of specificities and activation mechanisms. However, for the clostripain family (denoted C11), little is currently known. Here, we describe the first crystal structure of a C11 protein from the human gut bacterium, *Parabacteroides merdae* (PmC11), determined to 1.7-Å resolution. PmC11 is a monomeric cysteine peptidase that comprises an extended caspase-like α/β/α sandwich and an unusual C-terminal domain. It shares core structural elements with clan CD cysteine peptidases but otherwise structurally differs from the other families in the clan. These studies also revealed a well ordered break in the polypeptide chain at Lys^147^, resulting in a large conformational rearrangement close to the active site. Biochemical and kinetic analysis revealed Lys^147^ to be an intramolecular processing site at which cleavage is required for full activation of the enzyme, suggesting an autoinhibitory mechanism for self-preservation. PmC11 has an acidic binding pocket and a preference for basic substrates, and accepts substrates with Arg and Lys in P1 and does not require Ca^2+^ for activity. Collectively, these data provide insights into the mechanism and activity of PmC11 and a detailed framework for studies on C11 peptidases from other phylogenetic kingdoms.

## Introduction

Cysteine peptidases play crucial roles in the virulence of bacterial and other eukaryotic pathogens. In the MEROPS peptidase database (1), clan CD contains groups (or families) of cysteine peptidases that share some highly conserved structural elements (2). Clan CD families are typically described using the name of their archetypal, or founding, member and also given an identification number preceded by a “C,” to denote cysteine peptidase. Although seven families (C14 is additionally split into three subfamilies) have been described for this clan, crystal structures have only been determined from four: legumain (C13) (3), caspase (C14a) (4), paracaspase (C14b(P) (5), metacaspase (C14b(M) (6), gingipain (C25) (7), and the cysteine peptidase domain (CPD) of various toxins (C80) (8). No structural information is available for clostripain (C11), separase (C50), or PrtH-peptidase (C85).

Clan CD enzymes have a highly conserved His/Cys catalytic dyad and exhibit strict specificity for the P_1_ residue of their substrates. However, despite these similarities, clan CD forms a functionally diverse group of enzymes: the overall structural diversity between (and at times within) the various families provides these peptidases with a wide variety of substrate specificities and activation mechanisms. Several members are initially expressed as proenzymes, demonstrating self-inhibition prior to full activation (2).

The archetypal and arguably most notable family in the clan is that of the mammalian caspases (C14a), although clan CD members are distributed throughout the entire phylogenetic kingdom and are often required in fundamental biological processes (2). Interestingly, little is known about the structure or function of the C11 proteins, despite their widespread distribution (1) and its archetypal member, clostripain from *Clostridium histolyticum*, first reported in the literature in 1938 (9). Clostripain has been described as an arginine-specific peptidase with a requirement for Ca^2+^ (10) and loss of an internal nonapeptide for full activation; lack of structural information on the family appears to have prohibited further investigation.

As part of an ongoing project to characterize commensal bacteria in the microbiome that inhabit the human gut, the structure of C11 peptidase, PmC11, from *Parabacteroides merdae* was determined using the Joint Center for Structural Genomics (JCSG)^4^ HTP structural biology pipeline (11). The structure was analyzed, and the enzyme was biochemically characterized to provide the first structure/function correlation for a C11 peptidase.

## Experimental Procedures

Cloning, expression, purification, crystallization, and structure determination of PmC11 were carried out using standard JCSG protocols (11) as follows.

### 

#### 

##### Cloning

Clones were generated using the polymerase incomplete primer extension (PIPE) cloning method (12). The gene encoding PmC11 (SP5111E) was amplified by polymerase chain reaction (PCR) from *P. merdae* genomic DNA using *PfuTurbo* DNA polymerase (Stratagene), using I-PIPE primers that included sequences for the predicted 5′ and 3′ ends (shown below). The expression vector, pSpeedET, which encodes an amino-terminal tobacco etch virus protease-cleavable expression and purification tag (MGSDKIHHHHHHENLYFQ/G), was PCR amplified with V-PIPE (Vector) primers. V-PIPE and I-PIPE PCR products were mixed to anneal the amplified DNA fragments together. *Escherichia coli* GeneHogs (Invitrogen) competent cells were transformed with the I-PIPE/V-PIPE mixture and dispensed on selective LB-agar plates. The cloning junctions were confirmed by DNA sequencing. The plasmid encoding the full-length protein was deposited in the PSI:Biology Materials Repository at the DNASU plasmid repository (PmCD00547516). For structure determination, to obtain soluble protein using the PIPE, method the gene segment encoding residues Met^1^-Asn^22^ was deleted because these residues were predicted to correspond to a signal peptide using SignalP (13).

##### Protein Expression and Selenomethionine Incorporation

The expression plasmid for the truncated PmC11 construct was transformed into *E. coli* GeneHogs competent cells and grown in minimal media supplemented with selenomethionine and 30 μg ml^−1^ of kanamycin at 37 °C using a GNF fermentor (14). A methionine auxotrophic strain was not required as selenomethionine is incorporated via the inhibition of methionine biosynthesis (15, 16). Protein expression was induced using 0.1% (w/v) l-arabinose and the cells were left to grow for a further 3 h at 37 °C. At the end of the cell culture, lysozyme was added to all samples to a final concentration of 250 μg ml^−1^ and the cells were harvested and stored at −20 °C, until required. Primers used in this section are as follows: I-PIPE (forward): CTGTACTTCCAGGGCGAGACTCCGGAACCCCGGACAACCCGC; I-PIPE (reverse): AATTAAGTCGCGTTATTCATAAACTGCCTTATACCAGCCGAC; V-PIPE (forward): TAACGCGACTTAATTAACTCGTTTAAACGGTCTCCAGC; and V-PIPE (reverse): GCCCTGGAAGTACAGGTTTTCGTGATGATGATGATGAT.

##### Protein Purification for Crystallization

Cells were resuspended, homogenized, and lysed by sonication in 40 mm Tris (pH 8.0), 300 mm NaCl, 10 mm imidazole, and 1 mm Tris(2-carboxyethyl)phosphine hydrochloride (TCEP) (Lysis Buffer 1) containing 0.4 mm MgSO_4_ and 1 μl of 250 unit/μl^−1^ of benzonase (Sigma). The cell lysate was then clarified by centrifugation (32,500 × *g* for 25 min at 4 °C) before being passed over Ni^2+^-chelating resin equilibrated in Lysis Buffer 1 and washed in the same buffer supplemented with 40 mm imidazole and 10% (v/v) glycerol. The protein was subsequently eluted in 20 mm Tris (pH 8.0), 150 mm NaCl, 10% (v/v) glycerol, 1 mm TCEP, and 300 mm imidazole, and the fractions containing the protein were pooled.

To remove the His tag, PmC11 was exchanged into 20 mm Tris (pH 8.0), 150 mm NaCl, 30 mm imidazole, and 1 mm TCEP using a PD-10 column (GE Healthcare), followed by incubation with 1 mg of His-tagged tobacco etch virus protease per 15 mg of protein for 2 h at room temperature and subsequent overnight incubation at 4 °C. The sample was centrifuged to remove any precipitated material (13,000 × *g* for 10 min at 4 °C) and the supernatant loaded onto Ni^2+^-chelating resin equilibrated with 20 mm Tris (pH 8.0), 150 mm NaCl, 30 mm imidazole, and 1 mm TCEP and washed with the same buffer. The flow-through and wash fractions were collected and concentrated to 13.3 mg ml^−1^ using Amicon Ultra-15 5K centrifugal concentrators (Millipore).

##### Crystallization and Data Collection

PmC11 was crystallized using the nanodroplet vapor diffusion method using standard JCSG crystallization protocols (11). Drops were comprised of 200 nl of protein solution mixed with 200 nl of crystallization solution in 96-well sitting-drop plates, equilibrated against a 50-μl reservoir. Crystals of PmC11 were grown at 4 °C in mother liquor consisting of 0.2 m NH_4_H_2_PO_4_, 20% PEG-3350 (JCSG Core Suite I). Crystals were flash cooled in liquid nitrogen using 10% ethylene glycol as a cryoprotectant prior to data collection and initial screening for diffraction was carried out using the Stanford Automated Mounting system (17) at the Stanford Synchrotron Radiation Lightsource (SSRL, Menlo Park, CA). Single wavelength anomalous dispersion data were collected using a wavelength of 0.9793 Å, at the Advanced Light Source (ALS, beamline 8.2.2, Berkeley, CA) on an ADSC Quantum 315 CCD detector. The data were indexed and integrated with XDS (18) and scaled using XSCALE (18). The diffraction data were indexed in space group P2_1_ with *a* = 39.11, *b* = 108.68, *c* = 77.97 Å, and β = 94.32°. The unit cell contained two molecules in the asymmetric unit resulting in a solvent content of 39% (Matthews' coefficient (V*_m_*) of 2.4 Å^3^ Da^−1^).

##### Structure Determination

The PmC11 structure was determined by the single wavelength anomalous dispersion method using an x-ray wavelength corresponding to the peak of the selenium K edge. Initial phases were derived using the autoSHARP interface (19), which included density modification with SOLOMON (20). Good quality electron density was obtained at 1.7-Å resolution, allowing an initial model to be obtained by automated model building with ARP/wARP (21). Model completion and refinement were iteratively performed with COOT (22) and REFMAC (23, 24) to produce a final model with an *R*_cryst_ and *R*_free_ of 14.3 and 17.5%, respectively. The refinement included experimental phase restraints in the form of Hendrickson-Lattman coefficients, TLS refinement with one TLS group per molecule in the asymmetric unit, and NCS restraints. The refined structure contains residues 24–375 and 28–375 for the two molecules in the crystallographic asymmetric unit. Structural validation was carried using the JCSG Quality Control Server that analyzes both the coordinates and data using a variety of structural validation tools to confirm the stereochemical quality of the model (ADIT (25), MOLPROBITY (26), and WHATIF 5.0 (27)) and agreement between model and data (SGCHECK (28) and RESOLVE (29)). All of the main-chain torsion angles were in the allowed regions of the Ramachandran plot and the MolProbity overall clash score for the structure was 2.09 (within the 99th percentile for its resolution). The atomic coordinates and structure factors for PmC11 have been deposited in the Protein Data Bank (PDB) with the accession code 3UWS. Data collection, model, and refinement statistics are reported in Table 1.

**TABLE 1:** **Crystallographic statistics for PDB code 3UWS** Values in parentheses are for the highest resolution shell.

**Data collection**	
Wavelength (Å)	0.9793
Space group	P2_1_
Unit cell dimensions *a*, *b*, *c* (Å); β^°^	39.11, 108.68, 77.97; β = 94.32°
Resolution range (Å)	28.73–1.70 (1.79–1.70)
Unique reflections	70,913
*R*_merge_*^a^* on *I* (%)	10.2 (49.0)
*R*_meas_*^b^* on *I* (%)	11.0 (52.7)
*R*_pim_*^c^* on *I* (%)	4.1 (19.2)
*I*/σ*_I_*	15.6 (4.6)
Wilson B (Å^2^)	15.9
Completeness (%)	99.6 (99.8)
Multiplicity	7.3 (7.5)

**Model and refinement**	
Reflections (total/test)	70,883/3,577
*R*_cryst_/*R*_free_*^d^* (%)	14.3/17.5
No. protein residues/atoms	700/5612
No. of water/EDO molecules	690/7
ESU*^e^* based on *R*_free_ (Å)	0.095
B-values (Å^2^)	
Average isotropic B (overall)	20.0
Protein overall	18.8
All main/side chains	16.7/20.8
Solvent/EDO	29.4/35.6
RMSD*^g^*	
Bond lengths (Å)	0.01
Bond angles (°)	1.6
Ramachandran analysis (%)	
Favored regions	97.0
Allowed regions	3.0
Outliers	0.0

*^a^ R*_merge_ = Σ*_hkl_*Σ*_i_*|*I_i_*(*hkl*) − 〈*I*(*hkl*)〉|/Σ*_hkl_* Σ*_i_*(*hkl*).

*^b^ R*_meas_ = Σ*_hkl_*[N/(N-1)]^1/2^Σ*_i_*|*I_i_*(*hkl*) − 〈*I*(*hkl*)〉|/Σ*_hkl_*Σ*_i_I_i_*(*hkl*).

*^c^ R*_pim_ (precision-indicating *R*_merge_) = Σ*_hkl_*[(1/(*N*-1)]^1/2^ Σ*_i_*|*I_i_* (*hkl*) − 〈*I*(*hkl*)〉|/Σ*_hkl_*Σ*_i_ I_i_*(*hkl*) (43), where *n* is the multiplicity of reflection *hkl*, and *I_i_*(*hkl*) and 〈*I*(*hkl*)〉 are the intensity of the ith measurement and the average intensity of reflection hkl, respectively (44).

*^d^ R*_cryst_ and *R*_free_ = Σ‖*F*_obs_| − |*F*_calc_‖/Σ|*F*_obs_| for reflections in the working and test sets, respectively, where *F*_obs_ and *F*_calc_ are the observed and calculated structure-factor amplitudes, respectively. *R*_free_ is the same as *R*_cryst_ but for 5% of the total reflections chosen at random and omitted from structural refinement.

*^e^* ESU is the estimated standard uncertainties of atoms.

*^f^* The average isotropic B includes TLS and residual B components.

*^g^* RMSD, root-mean-square deviation.

##### Structural Analysis

The primary sequence alignment with assigned secondary structure was prepared using CLUSTAL OMEGA (30) and ALINE (31). The topology diagram was produced with TOPDRAW (32) and all three-dimensional structural figures were prepared with PyMol (33) with the electrostatic surface potential calculated with APBS (18) and contoured at ±5 kT/e. Architectural comparisons with known structures revealed that PmC11 was most structurally similar to caspase-7, gingipain-K, and legumain (PBD codes 4hq0, 4tkx, and 4aw9, respectively). The statistical significance of the structural alignment between PmC11 and both caspase-7 and gingipain-K is equivalent (Z-score of 9.2) with legumain giving a very similar result (Z-score of 9.1). Of note, the β-strand topology of the CDP domains of *Clostridium difficile* toxin B (family C80; TcdB; PDB code 3pee) is identical to that observed in the PmC11 β-sheet, but the Z-score from DaliLite was notably less at 7.6. It is possible that the PmC11 structure is more closely related to the C80 family than other families in clan CD, and appear to reside on the same branch of the phylogenetic tree based on structure (2).

##### Protein Production for Biochemical Assays

The PmCD00547516 plasmid described above was obtained from the PSI:Biology Materials Repository and used to generate a cleavage site mutant *PmC11^K147A^* and an active-site mutant *PmC11^C179A^* using the QuikChange Site-directed Mutagenesis kit (Stratagene) as per the manufacturer's instructions using the following primers: K147A mutant (forward): CAGAATAAGCTGGCAGCGTTCGGACAGGACG, and K147A mutant (reverse): CGTCCTGTCCGAACGCTGCCAGCTTATTCTG; C179A mutant (forward): CCTGTTCGATGCCGCCTACATGGCAAGC, and C179A mutant (reverse): GCTTGCCATGTAGGCGGCATCGAACAGG. The expression plasmids containing *PmC11* were transformed into *E. coli* BL21 Star (DE3) and grown in Luria-Bertani media containing 30 μg ml^−1^ of kanamycin at 37 °C until an optical density (600 nm) of ∼0.6 was reached. l-Arabinose was added to a final concentration of 0.2% (w/v) and the cells incubated overnight at 25 °C.

Compared with the protein production for crystallography, a slightly modified purification protocol was employed for biochemical assays. Initially, the cells were resuspended in 20 mm sodium phosphate (pH 7.5), 150 mm NaCl (Lysis Buffer 2) containing an EDTA-free protease inhibitor mixture (cOmplete, Roche Applied Science). Cells were disrupted by three passages (15 KPSI) through a One-Shot cell disruptor (Constant Systems) followed by centrifugation at 20,000 × *g* for 20 min at 4 °C. The supernatant was collected and sterile-filtered (0.2 μm) before being applied to a 5-ml HisTrap HP column (GE Healthcare) equilibrated in Lysis Buffer 2 containing 25 mm imidazole, and the protein was eluted in the same buffer containing 250 mm imidazole. The peak fractions were pooled and buffer exchanged into the assay buffer (20 mm Tris, 150 mm NaCl, pH 8.0) using a PD-10 column. When required, purified PmC11 was concentrated using Vivaspin 2 30-K centrifugal concentrators (Sartorius). Protein concentration was routinely measured using Bradford's reagent (Bio-Rad) with a BSA standard.

##### Fluorogenic Substrate Activity Assays

The release of the fluorescent group AMC (7-amino-4-methylcoumarin) from potential peptide substrates was used to assess the activity of PmC11. Peptidase activity was tested using 20 μg of PmC11 and 100 μm substrate (unless otherwise stated) in assay buffer to a final reaction volume of 200 μl and all samples were incubated (without substrate) at 37 °C for 16 h prior to carrying out the assay. The substrate and plate reader were brought to 37 °C for 20 min prior to the addition of the PmC11 and samples prepared without PmC11 were used as blanks (negative controls). The curves were plotted using the blank-corrected fluorescence units against the time of acquisition (in min). The assays were carried out in black 96-well flat-bottomed plates (Greiner). AMC fluorescence was measured using a PHERAstar FS plate reader (BMG Labtech) with excitation and emission wavelengths of 355 and 460 nm, respectively.

To investigate the substrate specificity of PmC11, substrates Z-GGR-AMC, Bz-R-AMC, Z-GP-AMC, Z-HGP-AMC, Ac-DEVD-AMC (all Bachem), BOC-VLK-AMC, and BOC-K-AMC (both PeptaNova) were prepared at 100 mm in 100% dimethyl sulfoxide. The amount of AMC (micromoles) released was calculated by generating an AMC standard curve (as described in Ref. 34) and the specific activity of PmC11 was calculated as picomoles of AMC released per min per mg of the protein preparation.

The reaction rates (*V*_max_) and *K_m_* values were determined for mutants PmC11^K147A^ and PmC11^C179A^ by carrying out the activity assay at varying concentrations of Bz-R-AMC between 0 and 600 μm. The blank-corrected relative fluorescence units were plotted against time (min) with ΔFU/T giving the reaction rate. The *K_m_* and *V*_max_ of PmC11 and PmC11^K147A^ against an R-AMC substrate were determined from the Lineweaver-Burk plot as described (34), calculated using GraphPad Prism6 software. All experiments were carried out in triplicate.

##### Effect of VRPR-FMK on PmC11

To test the effect of the inhibitor on the activity of PmC11, 25 μm Z-VRPR-FMK (100 mm stock in 100% dimethyl sulfoxide, Enzo Life Sciences), 20 μg of PmC11, 100 μm R-AMC substrate, 1 mm EGTA were prepared in the assay buffer and the activity assay carried out as described above. A gel-shift assay, to observe Z-VRPR-FMK binding to PmC11, was also set up using 20 μg of PmC11, 25 μm inhibitor, 1 mm EGTA in assay buffer. The reactions were incubated at 37 °C for 20 min before being stopped by the addition SDS-PAGE sample buffer. Samples were analyzed by loading the reaction mixture on a 10% NuPAGE BisTris gel using MES buffer.

##### Effect of Cations on PmC11

The enzyme activity of PmC11 was tested in the presence of various divalent cations: Mg^2+^, Ca^2+^, Mn^2+^, Co^2+^, Fe^2+^, Zn^2+^, and Cu^2+^. The final concentration of the salts (MgSO_4_, CaCl_2_, MnCl_2_, CoCl_2_, FeSO_4_, ZnCl_2_, and CuSO_4_) was 1 mm and the control was set up without divalent ions but with addition of 1 mm EGTA. The assay was set up using 20 mg of PmC11, 1 mm salts, 100 μm R-AMC substrate, and the assay buffer, and incubated at 37 °C for 16 h. The activity assay was carried out as described above.

##### Size Exclusion Chromatography

Affinity-purified PmC11 was loaded onto a HiLoad 16/60 Superdex 200 gel filtration column (GE Healthcare) equilibrated in the assay buffer. The apparent molecular weight of PmC11 was determined from calibration curves based on protein standards of known molecular weights.

##### Autoprocessing Profile of PmC11

Autoprocessing of PmC11 was evaluated by incubating the enzyme at 37 °C and removing samples at 1-h intervals from 0 to 16 h and placing into SDS-PAGE loading buffer to stop the processing. Samples were then analyzed on a 4–12% NuPAGE (Thermo Fisher) Novex BisTris gel run in MES buffer.

##### Autoprocessing Cleavage Site Analysis

To investigate whether processing is a result of intra- or inter-molecular cleavage, the PmC11^C179A^ mutant was incubated with increasing concentrations of activated PmC11 (0, 0.1, 0.2, 0.5, 1, 2, and 5 μg). The final assay volume was 40 μl and the proteins were incubated at 37 °C for 16 h in the PmC11 assay buffer. To stop the reaction, NuPAGE sample buffer was added to the protein samples and 20 μl was analyzed on 10% NuPAGE Novex BisTris gel using MES buffer. These studies revealed no apparent cleavage of PmC11^C179A^ by the active enzyme at low concentrations of PmC11 and that only limited cleavage was observed when the ratio of active enzyme (PmC11: PmC11^C179A^) was increased to ∼1:10 and 1:4.

## Results

### 

#### 

##### Structure of PmC11

**FIGURE 1.**
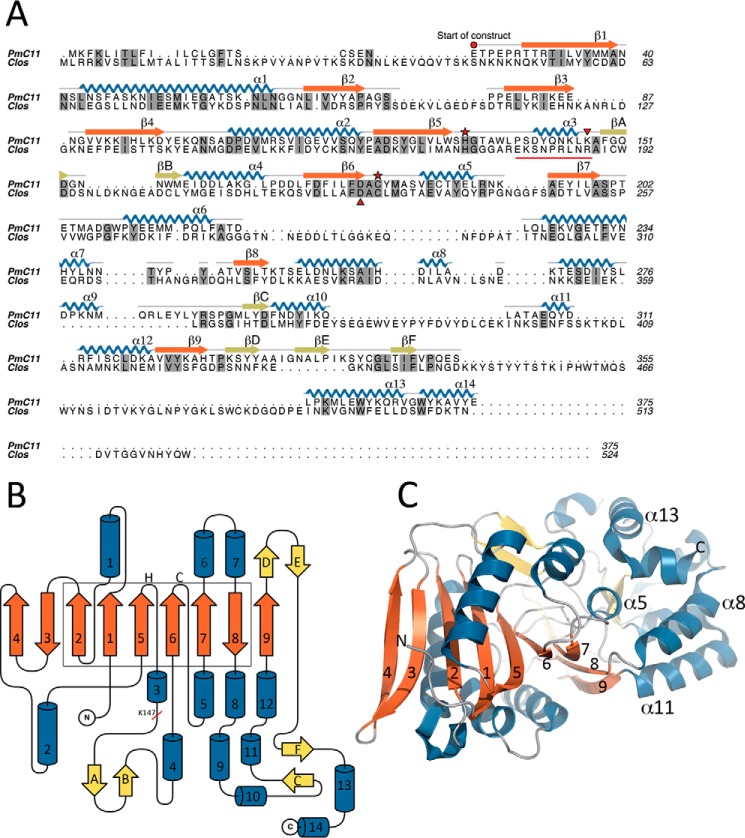
**Crystal structure of a C11 peptidase from *P. merdae*.** *A*, primary sequence alignment of PmC11 (Uniprot ID A7A9N3) and clostripain (Uniprot ID P09870) from *C. histolyticum* with identical residues highlighted in *gray shading*. The secondary structure of PmC11 from the crystal structure is mapped onto its sequence with the position of the PmC11 catalytic dyad, autocatalytic cleavage site (Lys^147^), and S_1_ binding pocket Asp (Asp^177^) highlighted by a *red star*, a *red downturned triangle*, and a *red upturned triangle*, respectively. Connecting loops are colored *gray*, the main β-sheet is in *orange*, with other strands in *olive*, α-helices are in *blue*, and the nonapeptide linker of clostripain that is excised upon autocleavage is *underlined in red*. Sequences around the catalytic site of clostripain and PmC11 align well. *B*, topology diagram of PmC11 colored as in *A* except that additional (non-core) β-strands are in *yellow*. Helices found on either side of the central β-sheet are shown above and below the sheet, respectively. The position of the catalytic dyad (*H*, *C*) and the processing site (Lys^147^) are highlighted. Helices (1–14) and β-strands (1–9 and *A-F*) are numbered from the N terminus. The core caspase-fold is highlighted in a *box. C,* tertiary structure of PmC11. The N and C termini (*N* and *C*) of PmC11 along with the central β-sheet (1–9), helix α5, and helices α8, α11, and α13 from the C-terminal domain, are all labeled. Loops are colored *gray*, the main β-sheet is in *orange*, with other β-strands in *yellow*, and α-helices are in *blue*.

The crystal structure of the catalytically active form of PmC11 revealed an extended caspase-like α/β/α sandwich architecture comprised of a central nine-stranded β-sheet, with an unusual C-terminal domain (CTD), starting at Lys^250^. A single cleavage was observed in the polypeptide chain at Lys^147^ (Fig. 1, *A* and *B*), where both ends of the cleavage site are fully visible and well ordered in the electron density. The central nine-stranded β-sheet (β1–β9) of PmC11 consists of six parallel and three anti-parallel β-strands with 4_↑_3_↓_2_↑_1_↑_5_↑_6_↑_7_↓_8_↓_9_↑_ topology (Fig. 1*A*) and the overall structure includes 14 α-helices with six (α1–α2 and α4–α7) closely surrounding the β-sheet in an approximately parallel orientation. Helices α1, α7, and α6 are located on one side of the β-sheet with α2, α4, and α5 on the opposite side (Fig. 1*A*). Helix α3 sits at the end of the loop following β5 (L5), just preceding the Lys^147^ cleavage site, with both L5 and α3 pointing away from the central β-sheet and toward the CTD, which starts with α8. The structure also includes two short β-hairpins (βA–βB and βD–βE) and a small β-sheet (βC–βF), which is formed from two distinct regions of the sequence (βC precedes α11, α12 and β9, whereas βF follows the βD-βE hairpin) in the middle of the CTD (Fig. 1*B*).

The CTD of PmC11 is composed of a tight helical bundle formed from helices α8–α14 and includes strands βC and βF, and β-hairpin βD–βE. The CTD sits entirely on one side of the enzyme interacting only with α3, α5, β9, and the loops surrounding β8. Of the interacting secondary structure elements, α5 is perhaps the most interesting. This helix makes a total of eight hydrogen bonds with the CTD, including one salt bridge (Arg^191^-Asp^255^) and is surrounded by the CTD on one side and the main core of the enzyme on the other, acting like a linchpin holding both components together (Fig. 1*C*).

##### Structural Comparisons

PmC11 is, as expected, most structurally similar to other members of clan CD with the top hits in a search of known structures being caspase-7, gingipain-K, and legumain (PBD codes 4hq0, 4tkx, and 4aw9, respectively) (Table 2). The C-terminal domain is unique to PmC11 within clan CD and structure comparisons for this domain alone does not produce any hits in the PDB (DaliLite, PDBeFold), suggesting a completely novel fold. As the archetypal and arguably most well studied member of clan CD, the caspases were used as the basis to investigate the structure/function relationships in PmC11, with caspase-7 as the representative member. Six of the central β-strands in PmC11 (β1–β2 and β5–β8) share the same topology as the six-stranded β-sheet found in caspases, with strands β3, β4, and β9 located on the outside of this core structure (2) (Fig. 1*B*, *box*). His^133^ and Cys^179^ were found at locations structurally homologous to the caspase catalytic dyad, and other clan CD structures (2), at the C termini of strands β5 and β6, respectively (Figs. 1, *A* and *B,* and 2*A*). A multiple sequence alignment of C11 proteins revealed that these residues are highly conserved (data not shown).

**TABLE 2:** **Summary of PDBeFOLD (45) superposition of structures found to be most similar to PmC11 in the PBD based on DaliLite (46)** The results are ordered in terms of structural homology (Q_H_), where %SSE^PC-X^ is the percentage of the SSEs in the PmC11 that can be identified in the target X (where X = caspase-7 (47), legumain (3), gingipain (48), and TcdB-CPD (49); % SSE^X-PC^ is the percentage of SSEs in X (as above) that can be identified in PmC11 (as above); % sequence ID is the percentage sequence identity after structural alignment; N_align_ is the number of matched residues; and r.m.s. deviation the root mean squared deviation on the Cα positions of the matched residues.

Enzyme	Family	PDB code	Q^H^	Z-score	%SSE^PC-X^	%SSE^X-PC^	% Seq. ID	*N*_align_	RMSD (Å)	*N*_Strands_
PmC11	C11	3UWS	1.00	33.4	100	100	100	352	0.00	9
Caspase-7	C14A	4HQ0	0.16	4.3	38	79	14	162	3.27	6
Legumain	C13	4AW9	0.13	5.5	31	53	13	161	2.05	6
TcdB-CPD	C80	3PEE	0.10	4.9	28	50	12	138	3.18	9
Gingipain	C25	4TKX	0.07	5.4	28	27	12	153	2.97	10

**FIGURE 2.**
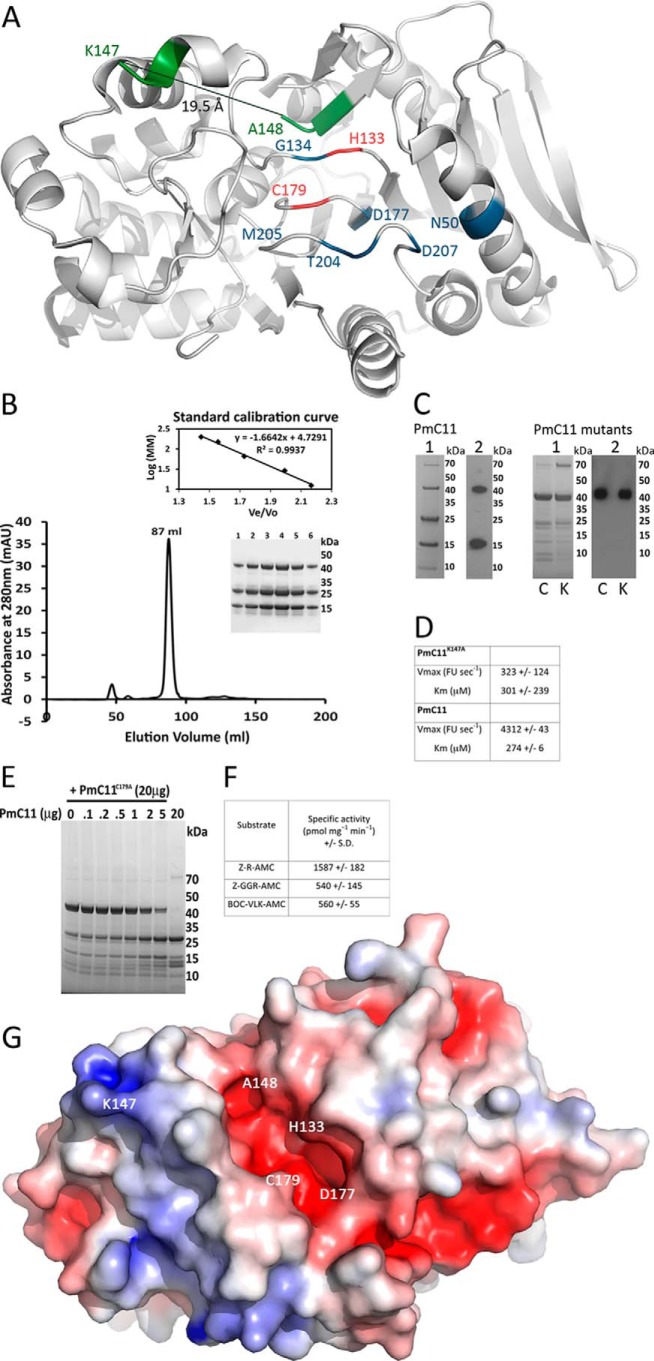
**Biochemical and structural characterization of PmC11.** *A,* ribbon representation of the overall structure of PmC11 illustrating the catalytic site, cleavage site displacement, and potential S_1_ binding site. The overall structure of PmC11 is shown in *gray*, looking down into the catalytic site with the catalytic dyad in *red*. The two ends of the autolytic cleavage site (Lys^147^ and Ala^148^, *green*) are displaced by 19.5 Å (*thin black line*) from one another and residues in the potential substrate binding pocket are highlighted in *blue. B,* size exclusion chromatography of PmC11. PmC11 migrates as a monomer with a molecular mass around 41 kDa calculated from protein standards of known molecular weights. Elution fractions across the major peak (1–6) were analyzed by SDS-PAGE on a 4–12% gel in MES buffer. *C*, the active form of PmC11 and two mutants, PmC11^C179A^ (*C*) and PmC11^K147A^ (*K*), were examined by SDS-PAGE (*lane 1*) and Western blot analysis using an anti-His antibody (*lane 2*) show that PmC11 autoprocesses, whereas mutants, PmC11^C179A^ and PmC11^K147A^, do not show autoprocessing *in vitro. D,* cysteine peptidase activity of PmC11. *K_m_* and *V*_max_ of PmC11 and K147A mutant were determined by monitoring change in the fluorescence corresponding to AMC release from Bz-R-AMC. Reactions were performed in triplicate and representative values ± S.D. are shown. *E,* intermolecular processing of PmC11^C179A^ by PmC11. PmC11^C179A^ (20 μg) was incubated overnight at 37 °C with increasing amounts of processed PmC11 and analyzed on a 10% SDS-PAGE gel. Inactive PmC11^C179A^ was not processed to a major extent by active PmC11 until around a ratio of 1:4 (5 μg of active PmC11). A single lane of 20 μg of active PmC11 (labeled 20) is shown for comparison. *F*, activity of PmC11 against basic substrates. Specific activity is shown ± S.D. from three independent experiments. *G,* electrostatic surface potential of PmC11 shown in a similar orientation, where *blue* and *red* denote positively and negatively charged surface potential, respectively, contoured at ±5 kT/e. The position of the catalytic dyad, one potential key substrate binding residue Asp^177^, and the ends of the cleavage site Lys^147^ and Ala^148^ are indicated.

Five of the α-helices surrounding the β-sheet of PmC11 (α1, α2, α4, α6, and α7) are found in similar positions to the five structurally conserved helices in caspases and other members of clan CD, apart from family C80 (2). Other than its more extended β-sheet, PmC11 differs most significantly from other clan CD members at its C terminus, where the CTD contains a further seven α-helices and four β-strands after β8.

##### Autoprocessing of PmC11

Purification of recombinant PmC11 (molecular mass = 42.6 kDa) revealed partial processing into two cleavage products of 26.4 and 16.2 kDa, related to the observed cleavage at Lys^147^ in the crystal structure (Fig. 2*A*). Incubation of PmC11 at 37 °C for 16 h, resulted in a fully processed enzyme that remained as an intact monomer when applied to a size-exclusion column (Fig. 2*B*). The single cleavage site of PmC11 at Lys^147^ is found immediately after α3, in loop L5 within the central β-sheet (Figs. 1, *A* and *B,* and 2A). The two ends of the cleavage site are remarkably well ordered in the crystal structure and displaced from one another by 19.5 Å (Fig. 2*A*). Moreover, the C-terminal side of the cleavage site resides near the catalytic dyad with Ala^148^ being 4.5 and 5.7 Å from His^133^ and Cys^179^, respectively. Consequently, it appears feasible that the helix attached to Lys^147^ (α3) could be responsible for steric autoinhibition of PmC11 when Lys^147^ is covalently bonded to Ala^148^. Thus, the cleavage would be required for full activation of PmC11. To investigate this possibility, two mutant forms of the enzyme were created: PmC11^C179A^ (a catalytically inactive mutant) and PmC11^K147A^ (a cleavage-site mutant). Initial SDS-PAGE and Western blot analysis of both mutants revealed no discernible processing occurred as compared with active PmC11 (Fig. 2*C*). The PmC11^K147A^ mutant enzyme had a markedly different reaction rate (*V*_max_) compared with WT, where the reaction velocity of PmC11 was 10 times greater than that of PmC11^K147A^ (Fig. 2*D*). Taken together, these data reveal that PmC11 requires processing at Lys^147^ for optimum activity.

To investigate whether processing is a result of intra- or intermolecular cleavage, the PmC11^C179A^ mutant was incubated with increasing concentrations of processed and activated PmC11. These studies revealed that there was no apparent cleavage of PmC11^C179A^ by the active enzyme at low concentrations of PmC11 and that only limited cleavage was observed when the ratio of active enzyme (PmC11:PmC11^C179A^) was increased to ∼1:10 and 1:4, with complete cleavage observed at a ratio of 1:1 (Fig. 2*E*). This suggests that cleavage of PmC11^C179A^ was most likely an effect of the increasing concentration of PmC11 and intermolecular cleavage. Collectively, these data suggest that the pro-form of PmC11 is autoinhibited by a section of L5 blocking access to the active site, prior to intramolecular cleavage at Lys^147^. This cleavage subsequently allows movement of the region containing Lys^147^ and the active site to open up for substrate access.

##### Substrate Specificity of PmC11

The autocatalytic cleavage of PmC11 at Lys^147^ (sequence KLK∧A) demonstrates that the enzyme accepts substrates with Lys in the P_1_ position. The substrate specificity of the enzyme was further tested using a variety of fluorogenic substrates. As expected, PmC11 showed no activity against substrates with Pro or Asp in P_1_ but was active toward substrates with a basic residue in P_1_ such as Bz-R-AMC, Z-GGR-AMC, and BOC-VLK-AMC. The rate of cleavage was ∼3-fold greater toward the single Arg substrate Bz-R-AMC than for the other two (Fig. 2*F*) and, unexpectedly, PmC11 showed no activity toward BOC-K-AMC. These results confirm that PmC11 accepts substrates containing Arg or Lys in P_1_ with a possible preference for Arg.

The catalytic dyad of PmC11 sits near the bottom of an open pocket on the surface of the enzyme at a conserved location in the clan CD family (2). The PmC11 structure reveals that the catalytic dyad forms part of a large acidic pocket (Fig. 2*G*), consistent with a binding site for a basic substrate. This pocket is lined with the potential functional side chains of Asn^50^, Asp^177^, and Thr^204^ with Gly^134^, Asp^207^, and Met^205^ also contributing to the pocket (Fig. 2*A*). Interestingly, these residues are in regions that are structurally similar to those involved in the S_1_ binding pockets of other clan CD members (shown in Ref. 2).

**FIGURE 3.**
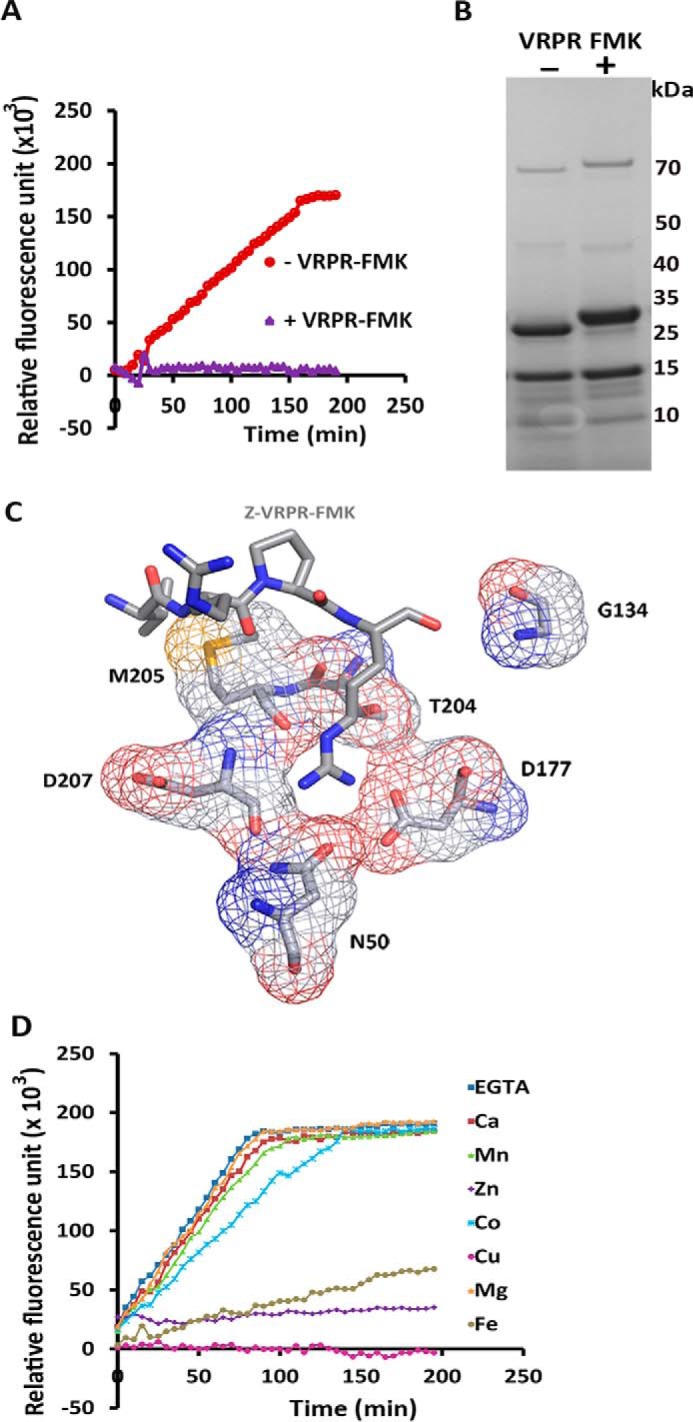
**PmC11 binds and is inhibited by Z-VRPR-FMK and does not require Ca^2+^ for activity.** *A*, PmC11 activity is inhibited by Z-VRPR-FMK. Cleavage of Bz-R-AMC by PmC11 was measured in a fluorometric activity assay with (+, *purple*) and without (−, *red*) Z-VRPR-FMK. The relative fluorescence units of AMC released are plotted against time (min) (*n* = 3; ±S.D.). *B*, gel-shift assay reveals that Z-VRPR-FMK binds to PmC11. PmC11 was incubated with (+) or without (−) Z-VRPR-FMK and the samples analyzed on a 10% SDS-PAGE gel. A size shift can be observed in the larger processed product of PmC11 (26.1 kDa). *C*, PmC11 with the Z-VRPR-FMK from the MALT1-paracacaspase (MALT1-P) superimposed. A three-dimensional structural overlay of Z-VRPR-FMK from the MALT1-P complex onto PmC11. The position and orientation of Z-VRPR-FMK was taken from superposition of the PmC11 and MALTI_P structures and indicates the presumed active site of PmC11. Residues surrounding the inhibitor are labeled and represent potentially important binding site residues, labeled in *black* and shown in an atomic representation. Carbon atoms are shown in *gray*, nitrogen in *blue*, and oxygen in *red. C,* divalent cations do not increase the activity of PmC11. The cleavage of Bz-R-AMC by PmC11 was measured in the presence of the cations Ca^2+^, Mn^2+^, Zn^2+^, Co^2+^, Cu^2+^, Mg^2+^, and Fe^3+^ with EGTA as a negative control, and relative fluorescence measured against time (min). The addition of cations produced no improvement in activity of PmC11 when compared in the presence of EGTA, suggesting that PmC11 does not require metal ions for proteolytic activity. Furthermore, Cu^2+^, Fe^2+^, and Zn^2+^ appear to inhibit PmC11.

Because PmC11 recognizes basic substrates, the tetrapeptide inhibitor Z-VRPR-FMK was tested as an enzyme inhibitor and was found to inhibit both the autoprocessing and activity of PmC11 (Fig. 3*A*). Z-VRPR-FMK was also shown to bind to the enzyme: a size-shift was observed, by SDS-PAGE analysis, in the larger processed product of PmC11 suggesting that the inhibitor bound to the active site (Fig. 3*B*). A structure overlay of PmC11 with the MALT1-paracacaspase (MALT1-P), in complex with Z-VRPR-FMK (35), revealed that the PmC11 dyad sits in a very similar position to that of active MALT1-P and that Asn^50^, Asp^177^, and Asp^207^ superimpose well with the principal MALT1-P inhibitor binding residues (Asp^365^, Asp^462^, and Glu^500^, respectively (VRPR-FMK from MALT1-P with the corresponding PmC11 residues from the structural overlay is shown in Fig. 1*D*), as described in Ref. 5). Asp^177^ is located near the catalytic cysteine and is conserved throughout the C11 family, suggesting it is the primary S_1_ binding site residue. In the structure of PmC11, Asp^207^ resides on a flexible loop pointing away from the S_1_ binding pocket (Fig. 3*C*). However, this loop has been shown to be important for substrate binding in clan CD (2) and this residue could easily rotate and be involved in substrate binding in PmC11. Thus, Asn^50^, Asp^177^, and Asp^207^ are most likely responsible for the substrate specificity of PmC11. Asp^177^ is highly conserved throughout the clan CD C11 peptidases and is thought to be primarily responsible for substrate specificity of the clan CD enzymes, as also illustrated from the proximity of these residues relative to the inhibitor Z-VRPR-FMK when PmC11 is overlaid on the MALT1-P structure (Fig. 3*C*).

##### Comparison with Clostripain

Clostripain from *C. histolyticum* is the founding member of the C11 family of peptidases and contains an additional 149 residues compared with PmC11. A multiple sequence alignment revealed that most of the secondary structural elements are conserved between the two enzymes, although they are only ∼23% identical (Fig. 1*A*). Nevertheless, PmC11 may be a good model for the core structure of clostripain.

The primary structural alignment also shows that the catalytic dyad in PmC11 is structurally conserved in clostripain (36) (Fig. 1*A*). Unlike PmC11, clostripain has two cleavage sites (Arg^181^ and Arg^190^), which results in the removal of a nonapeptide, and is required for full activation of the enzyme (37) (highlighted in Fig. 1*A*). Interestingly, Arg^190^ was found to align with Lys^147^ in PmC11. In addition, the predicted primary S_1_-binding residue in PmC11 Asp^177^ also overlays with the residue predicted to be the P1 specificity determining residue in clostripain (38) (Asp^229^, Fig. 1*A*).

As studies on clostripain revealed addition of Ca^2+^ ions are required for full activation, the Ca^2+^ dependence of PmC11 was examined. Surprisingly, Ca^2+^ did not enhance PmC11 activity and, furthermore, other divalent cations, Mg^2+^, Mn^2+^, Co^2+^, Fe^2+^, Zn^2+^, and Cu^2+^, were not necessary for PmC11 activity (Fig. 3*D*). In support of these findings, EGTA did not inhibit PmC11 suggesting that, unlike clostripain, PmC11 does not require Ca^2+^ or other divalent cations, for activity.

## Discussion

The crystal structure of PmC11 now provides three-dimensional information for a member of the clostripain C11 family of cysteine peptidases. The enzyme exhibits all of the key structural elements of clan CD members, but is unusual in that it has a nine-stranded central β-sheet with a novel C-terminal domain. The structural similarity of PmC11 with its nearest structural neighbors in the PDB is decidedly low, overlaying better with six-stranded caspase-7 than any of the other larger members of the clan (Table 2). The substrate specificity of PmC11 is Arg/Lys and the crystal structure revealed an acidic pocket for specific binding of such basic substrates. In addition, the structure suggested a mechanism of self-inhibition in both PmC11 and clostripain and an activation mechanism that requires autoprocessing. PmC11 differs from clostripain in that is does not appear to require divalent cations for activation.

Several other members of clan CD require processing for full activation including legumain (39), gingipain-R (40), MARTX-CPD (8), and the effector caspases, *e.g.* caspase-7 (41). To date, the effector caspases are the only group of enzymes that require cleavage of a loop within the central β-sheet. This is also the case in PmC11, although the cleavage loop is structurally different to that found in the caspases and follows the catalytic His (Fig. 1*A*), as opposed to the Cys in the caspases.

All other clan CD members requiring cleavage for full activation do so at sites external to their central sheets (2). The caspases and gingipain-R both undergo intermolecular (*trans*) cleavage and legumain and MARTX-CPD are reported to perform intramolecular (*cis*) cleavage. In addition, several members of clan CD exhibit self-inhibition, whereby regions of the enzyme block access to the active site (2). Like PmC11, these structures show preformed catalytic machinery and, for a substrate to gain access, movement and/or cleavage of the blocking region is required.

The structure of PmC11 gives the first insight into this class of relatively unexplored family of proteins and should allow important catalytic and substrate binding residues to be identified in a variety of orthologues. Indeed, insights gained from an analysis of the PmC11 structure revealed the identity of the *Trypanosoma brucei* PNT1 protein as a C11 cysteine peptidase with an essential role in organelle replication (42). The PmC11 structure should provide a good basis for structural modeling and, given the importance of other clan CD enzymes, this work should also advance the exploration of these peptidases and potentially identify new biologically important substrates.

## Author Contributions

K. M., J. S. G., D. D., I. A. W., and J. C. M. designed the research; K. M., J. S. G., and D. D. performed the research; K. M., J. S. G., D. D., G. H. C., A. S., M. A. E., and J. C. M. analyzed the data; A. G., S. A. L., A. M. D., M. A. E., and I. A. W. supervised various components of the JCSG structural genomics pipeline; M. K. G., A. G., S. A. L., A. M. D., and M. A. E. contributed reagents, materials, and analysis tools; and K. M., J. S. G., G. H. C., M. A. E., I. A. W., and J. C. M. wrote the paper.

## Footnotes

4 The abbreviations used are:

JCSG: Joint Center for Structural Genomics
PIPE: polymerase incomplete primer extension
TCEP: Tris(2-carboxyethyl)phosphine
AMC: 7-amino-4-methylcoumarin
PDB: Protein Data Bank
BisTris: 2-[bis(2-hydroxyethyl)amino]-2-(hydroxymethyl)propane-1,3-diol
Z: benzyloxycarbonyl
FMK: fluoromethyl ketone
CTD: C-terminal domain
Bz-R-AMC: benzoyl-l-Arg-4-methylcoumaryl-7-amide
Z-GGR-AMC: benzyloxycarbonyl-Gly-Gly-Arg-AMC
BOC-VLK-AMC: *t*-butyloxycarbonyl-Val-Leu-Lys.
